# Evaluating the efficacy of Nd:YAG fourth harmonic (266 nm) in comparison with ArF excimer (193 nm) in laser corneal reshaping: ex vivo pilot study

**DOI:** 10.1007/s10792-023-02708-z

**Published:** 2023-04-21

**Authors:** Ibrahim Abdelhalim, Omnia Hamdy, Mohamed A. Khattab, Salwa Abdelkawi, Salah Hassab Elnaby, Aziza Ahmed Hassan

**Affiliations:** 1 Engineering Applications of Laser Department, The National Institute of Laser Enhanced Sciences, Cairo University, Giza, 12613 Egypt; 2 Department of Cytology and Histology, Faculty of Veterinary Medicine, Cairo University, Giza, 12211 Egypt; 3 Vision Science Department, Biophysics and Laser Science Unit, Research Institute of Ophthalmology, Giza, Egypt; 4 Medical Applications of Laser Department, The National Institute of Laser Enhanced Sciences, Cairo University, Giza, 12613 Egypt

**Keywords:** Corneal reshaping, Laser ablation, DNA damage

## Abstract

**Purpose:**

Laser corneal reshaping is a common eye surgery utilized to overcome many vision disorders. Different UV laser wavelengths can be effective in the treatment. However, the ArF excimer laser (193 nm) is the most commonly used due to its high absorption in the cornea. In the current study, we investigate the efficacy of applying a solid-state laser (Nd:YAG fourth harmonic at 266 nm) for the corneal reshaping procedure.

**Methods:**

The utilized laser is generated using an optical setup based on a BBO nonlinear crystal which converts the Q-switched laser (532 nm) to its fourth harmonic (266 nm). Different pulse energies were applied with the same number of the shoots on ex vivo rabbit corneas, and the histological effect is studied. Moreover, the possible thermal damage on the treated corneal tissues was inspected via electron microscope. Additionally, the DNA damage on the corneal cells due to the application of the proposed laser was examined and compared with the existing technology (ArF Excimer laser at 193 nm) using the comet assay.

**Results:**

The histological examination revealed an appropriate ablation result with the minimum thermal effect at 1.5 mJ and 2.0 mJ. The overall results show that applying 50-shoots of the 1.5-mJ pulse energy using the proposed 266-nm solid-state laser produces the optimum ablation effect with the minimum thermal damage, and almost the same DNA damage occurred using the commercial 193-nm ArF excimer laser.

**Conclusion:**

Solid-state laser at 266 nm could be a good alternative to the common 193-nm excimer laser for corneal reshaping procedures.

**Supplementary Information:**

The online version contains supplementary material available at 10.1007/s10792-023-02708-z.

## Introduction

Lasers have been significantly influenced in various medical fields, including dermatology [1–3], surgery [3, 4], dentistry [5] and ophthalmology [6, 7]. They are widely utilized in different diagnostics and therapeutic applications such as optical imaging [8, 9], photodynamic therapy [10] and refractive eye surgeries [11]. In refractive eye surgery, a specific layer of the cornea named “stroma” is reshaped to correct the vision according to the occurred disorder [12]. Because the cornea is responsible for more than 65% of the total refractive power of the human eye, any distortion in the curvature of the cornea affects the total refractive power of the eye [13]. Therefore, predefined laser pulses are applied to reshape the stroma to correct common vision disorders like myopia, hyperopia and astigmatism via the laser photoablation effect [14].

Commonly, UV pulsed lasers with a nanosecond pulse duration are utilized for the corneal reshaping process due to the strong absorbance by the main chromophore in the cornea (that is, the polypeptide bond between the amino acids in the collagen fibers) at that spectral range [15–17].

Because of its adequate photon energy (6.4 eV) and precision, the cornea's proteins, glycosaminoglycans and nucleic acids are significantly absorbed by the laser wavelength 193 nm. As a consequence, it is found in the vast majority of industrial laser eye surgery equipments. Although the argon fluoride (ArF) excimer is the most commonly used gas laser [18], it has some drawbacks including a hazardous active medium, a risky electrical pumping method, complicated maintenance and a high cost [19, 20]. Therefore, different types of lasers in the UV range have been utilized in such a procedure. including krypton chloride KCL (223 nm) and krypton fluoride (KF) (248 nm). Moreover, the fourth harmonic of Ti: sapphire (210 nm) and fifth harmonic Nd:YAG (213 nm) are examples of UV solid-state lasers that have also been proposed for the corneal procedure [12, 21]. Solid-state lasers use no harmful gases and have a minimal power requirement, in addition to the diminished thermal effect on the corneal tissue [22]. Moreover, the laser parameters such as pulse duration, spot size and pulse energy are the key factors required for achieving the appropriate ablation effect with the minimum thermal damage [23].

The current study investigates the photoablation effect on ex vivo rabbit cornea after applying the same number of laser pulses (fourth harmonic Nd: YAG at 266 nm) with different pulse energies. Moreover, the resultant thermal damage to the cornea has been evaluated under the electron microscope and histological investigations. Furthermore, the arisen DNA damage on the corneal tissue using the solid-state laser (266 nm) is compared with that obtained using the standard gas laser (ArF excimer at 193) via comet assay.

## Materials and method

### Laser generation

The utilized laser source in the present study is pulsed Q-switched 532-nm second harmonic generation of Nd: YAG (1064 nm) with pulse duration 3–5 ns, pulse energy 14 mJ and spot size 3 mm. To generate the 266-nm laser, the 532-nm laser is directed to 4 × 4 × 10 mm^3^ BBO crystal with *θ* = 47.7° and Ф = 0, where *θ* is the angle between the incident wave vector and optic axis of the crystal and Ф is angle between projection of incident wave vector on the *x*–*y* plane and the *x* axis of the crystal principle axis. The crystal can be rotated to control the energy output per pulse; then, the beam is directed to the harmonic separator placed at 45° to the laser beam to reflect the 266-nm laser and transmits the other wavelengths (532 nm and 1064 nm).

**Fig. 1:**
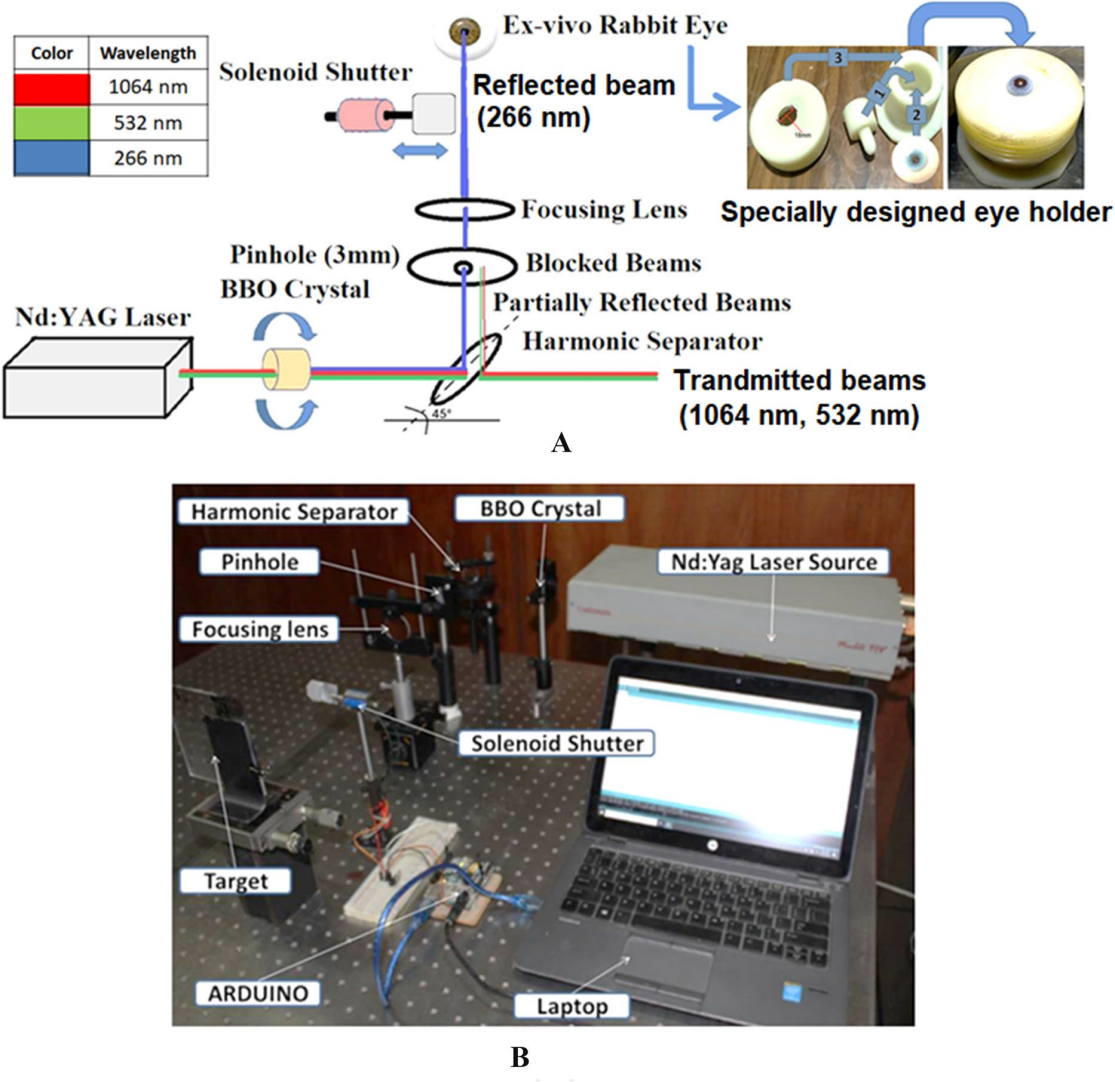
**a** Schematic diagram of the optical setup utilized for laser generation, **b** laboratory photograph of the constructed experimental setup [23]

The 266-nm laser is reflected toward a pinhole to block the partially reflected beams from the harmonic separator and transmits the 266 nm only. Then the laser beam is focused by a fused silica lens with a focal length of 300 mm. The spot size of the produced UV laser beam (266 nm) is 0.4 × 0.7 mm^2^, and the energy stability is ± 1%. Additionally, the beam spatial profile (fit to Gaussian) is 0/95. The resultant laser pulse on the focal point is ellipse-shaped spot with 2.5 mJ maximum pulse energy [23]. The experimental configuration is presented in Fig. 1.

The number of pulses (i.e., dose) is selected by a solenoid shutter that is controlled by ARDUINO platform to get the required number of pulses on the sample surface. The performance of the solenoid shutter system was tested and calibrated using polymethylmethacrylate (PMMA) targets before performing the ex vivo cornea measurements to avoid any possible errors on the treatment results. (Supplementary material S1 presents the implemented Arduino code for controlling the number of laser pulses has.)

### Ex vivo sample collection and preparation

The ex vivo samples (total 10 eyes collected from 5 rabbits) were purchased from local butcheries within one hour from slaughtering. Mechanical epithelium debridement of the central 7.5 mm of the cornea (previously marked with a 7.5 mm trephine) was performed. The eye samples were then placed on a specially designed holder for whole eye globe fixation (see Fig. 1). For investigating the histopathologic changes in the ex vivo rabbits’ cornea resulting from the ablation process, eye samples were flushed and fixed in 10% neutral buffered formalin for 72 h. Samples were then trimmed, processed in serial grades of ethanol and cleared in xylene. Furthermore, eye samples were infiltrated and embedded into Paraplast tissue embedding media. 4-μm-thick sections were sliced by rotatory microtome and mounted on glass slides.

### Laser ablation process

The eye samples were subjected to simultaneous laser shoots with different energies to asses the thermal effect using the histological examination. The studied samples were exposed to 1.5 mJ and 2 mJ (266-nm laser, 50 pulses) and were selected for the electron microscope examination to detect the thermal effect compared with the control sample (i.e., a native cornea before performing the laser ablation procedure).

The corneal samples were collected and fixed with osmium tetroxide and glutaraldehyde, then dehydrated in different concentrations of alcohol and embedded in the selected epoxy resin using a Leica Ultracut ultramicrotome UCT (Leica Microsystems, Germany); ultrathin sections were prepared at approximately 75–90 μm thickness and stained with lead citrate and uranyl acetate. Sections were examined and micrographed using transmission electron microscope (JEM-1400, Jeol, USA) having 0.38 nm point-to-point resolution. All the experimental procedures and animal protocols were approved by Cairo University—the Institutional Animal Care and Use Committee (CU-IACUC) with approval number: CU/I/F/9/22).

### Comet assay for measuring DNA damage

The comet assay is a simple and sensitive gel electrophoresis method that can be held to measure DNA damage from an individual cell. DNA damage of corneas was assessed using comet assay according to Olive and Banáth, 2006. Corneas were ground in phosphate buffer saline (Ca2 +—and Mg2 + -free) to form a single-cell suspension and kept in ice-cold medium. Using a hemocytometer, the concentration was adjusted to 2 × 105 cell/ml. Agarose-precoated slides were prepared by covering the slides with a thin layer of 1% low-gelling-temperature agarose (Sigma; Type VII, Cat. No. A-4018) and allowed to dry. The cornea cells suspension (0.4 ml) was mixed with 1% low-gelling-temperature agarose (1.2 ml) at 40 °C. Then, 1.2 ml from the mixture was spread on the precoated agarose slides and allowed to solidify.

Cell lysis is performed (to detect both single- and double-strand breaks in the DNA) in a covered jar using a cold fresh alkaline solution (1.2 M NaCl, 100 mM Na2EDTA, 0.1% sodium lauryl sarcosinate, 0.26 M NaOH, pH > 13) and equilibrate at 4 °C overnight (18–20 h) in the dark. The slides were removed and rinsed three times with alkaline rinse solution (0.03 M NaOH, 2 mM Na2EDTA (pH ∼ 12.3) for 20 min. The slides were immersed in the electrophoresis chamber containing the same rinse buffer solution. Electrophoresis was applied for 25 min at a voltage of 0.7 V/cm, 40 mA and 20 V. The slides were removed, rinsed with distilled water and stained with propidium iodide solution (2.5 µg/ml) for 20 min.

The DNA comets were visualized using a Nikon Optiphot-2 epifluorescence microscope (Plan Fluor, Nikon, UK) with an attached camera (Sony CCD-IRIS, Minato, Tokyo, Japan) and connected to a Pentium 1133 MHZ PC, which provided images on the Comet assay II software (Perceptive Instruments, UK). The software analyzes comet images for DNA content, tail length, percent DNA in tail and tail moment. Duplicate repeat experiments are recommended.

In the present study, cornea samples were subjected to 50 shoots of the produced 266-nm laser at five energies 0.5, 0.7, 1, 1.5 and 2 mJ to detect the DNA. In addition, another group of samples was subjected to the 193-nm laser with 1.0 mJ pulse energy, 4–7 ns pulse duration and 0.7 mm spot size (with an optical zone 6 mm). This sample group was divided into two groups: The first one was exposed to laser at 50 µm depth, while the second group at 100 µm depth (these depths are selected due to the limitations in the available commercial 193-nm excimer laser used for corneal reshaping) [24].

## Results and discussions

### The histological investigations

**Fig. 2:**
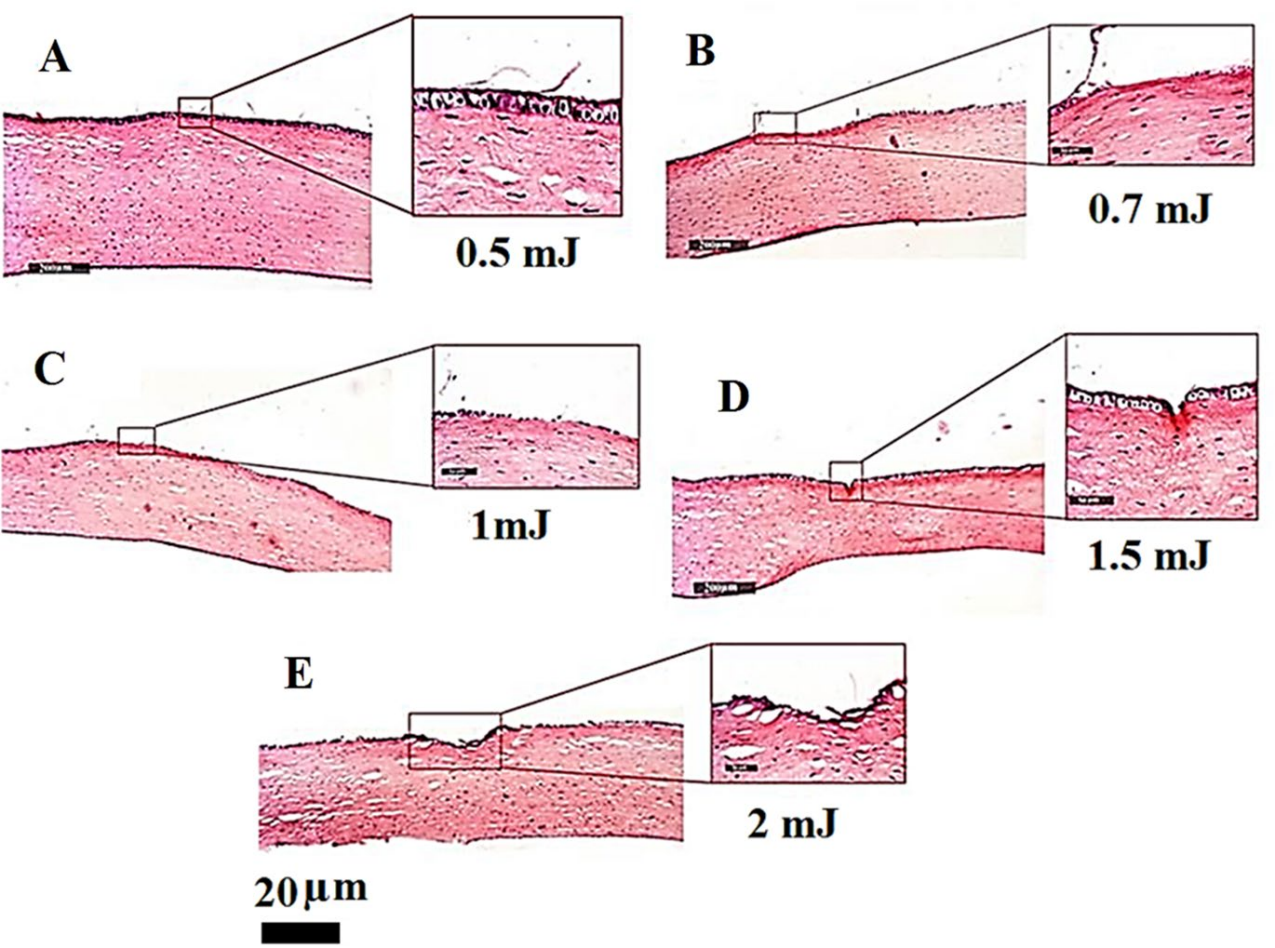
**a** Pulse energy 0.5 mJ, fluence 277 mJ/cm^2^; **b** pulse energy 0.7 mJ, fluence 318 mJ/cm^2^; **c** pulse energy 1.0 mJ, fluence 454.7 mJ/cm^2^; **d** pulse energy 1.5 mJ, fluence 682 mJ/cm^2^ **e** pulse energy 2 m, fluence 909 mJ/cm^2^

The aim of the present study was to select the best pulse energy of high photoablation with minimum thermal effect. Accordingly, histological examination has been performed using optical microscope (Lecia Microsystems GmbH, Germany). The histological analysis of the corneal samples exposed to different pulse energies showed the relation between the different pulse energies and the resultant ablation and thermal effect (Fig. 2).

The obtained ablation and thermal effect are summarized in Table 1. The 0.5 mJ pulse energy showed no ablation or thermal effect while the 0.7 mJ pulse energy showed the start of the photoablation effect with very weak effect and no thermal effect. The 1 mJ pulse energy showed photoablation effect and thermal effect the same as 0.7 mJ. Moreover, the 1.5 mJ pulse energy showed a good photoablation effect and obvious thermal effect.

**Table 1:** Resultant corneal ablation and thermal effect after 50 shoots from the 266-nm laser with different pulse energies and fluences

Pulse energy (mJ)	Fluence (mJ/mm^2)^	Ablation	Thermal effect
0.5	2.77	–	–
0.7	3.18	Very weak	–
1.0	5.47	Very weak	–
1.5	6.82	Good	–
2.0	9.09	High	Obvious

The exact mechanisms underlying UV laser ablation are still under exploration because the interaction of UV laser light with solid matter is a very complicated phenomenon. It is the result of the intricate interaction of several overlapping and distinct events, such as melting, electronic excitation, rapid heating and absorption [25]. For effective photoablation effect, the wavelength of the laser pulse should be selected to ensure that as much of it as possible is absorbed by the tissue being treated. Moreover, the laser pulse duration should be kept below the amount of time required for heat to diffuse out of the volume of the irradiated tissue to minimize thermal damage to the tissue surrounding the ablation crater [26].

Due to their high absorption in corneal tissue without affecting other areas of the eye, UV pulsed lasers are used in refractive surgeries. Since heat is the adversary of soft tissues, ablation should ideally not be connected to heat transfer. However, the wavelength-based corneal tissue absorption coefficient determines the heating impact during corneal reshaping. Lower absorption coefficient results in reduced temperature records at the corneal surface [12, 27]. The corneal tissue’s absorbance (A) at different UV wavelength has been thoroughly provided in [28]. Accordingly, the linear absorption coefficient (*α*) can be calculated as *α* = 2.303 A/d, where d is the thickness of the examined sample. Consequently, the corneal tissue’s absorption coefficient is 270,000 m^−1^ and 48,277 m^−1^ at 193 nm and 266 nm, repectively. Therefore, the thermal effect of the corneal ablation process using the 266-nm laser is lower than the 193-nm laser.

### Electron microscope examinations

**Fig. 3:**
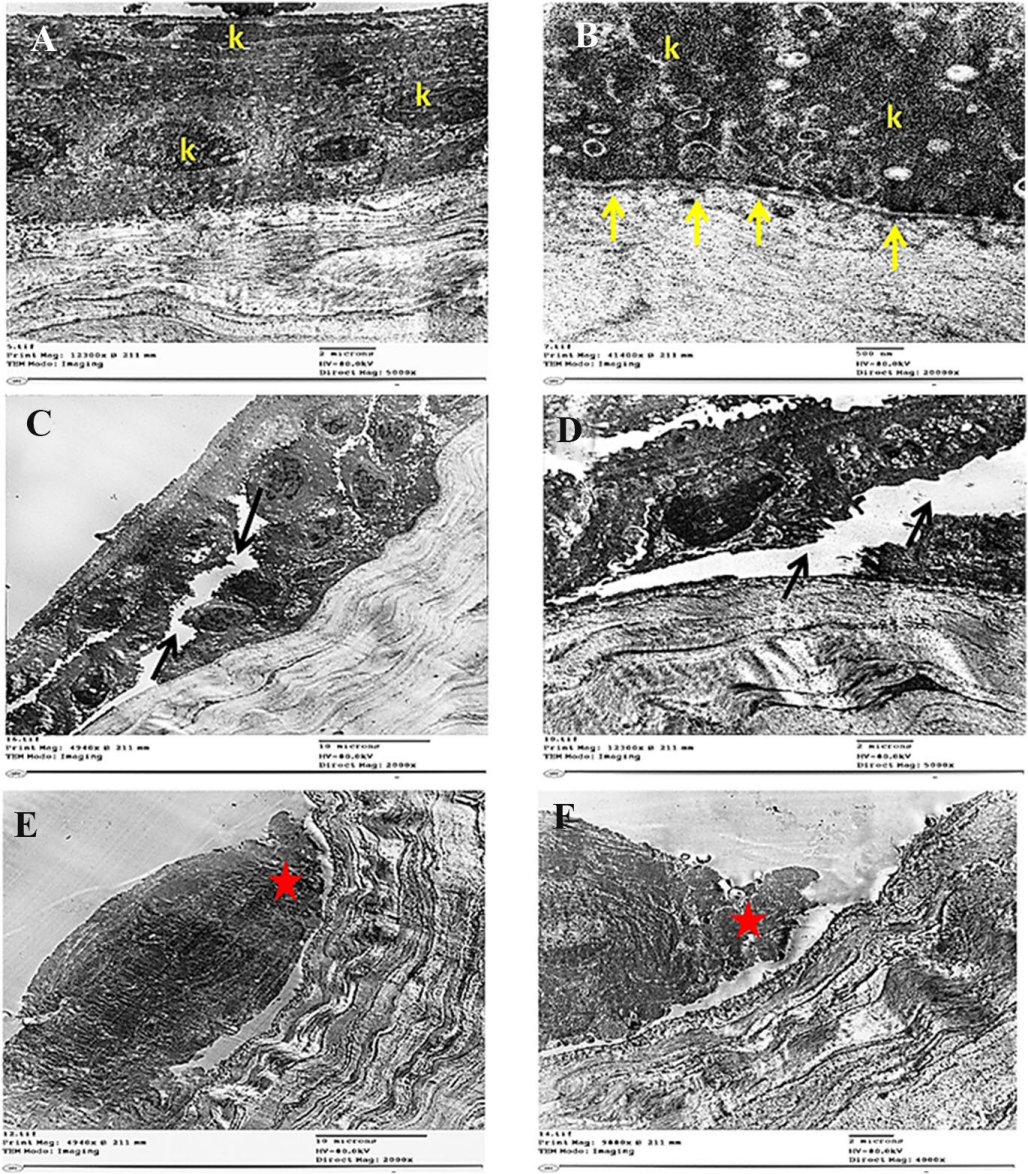
Transmission electron micrographs of different groups. Normal control (**a, b**), low energy 1.5 mJ (**c**, **d**) and high energy 2.0 mJ (**e**, **f**). **k** Keratenocytes, hemidesmosomes (yellow arrows), intracorneal cracks (black arrow), thermal damage (red star)

Two samples were exposed to 1.5 mJ and 2 mJ (266-nm laser, 50 pulses) irradiation, and the resultant thermal effect compared to the control sample was examined using electron microscope. The obtained results are presented in Fig. 3.

As demonstrated in the figure, normal control corneal sample revealed well-organized intact and interdigitating corneal keratinocytes with superficial flat smooth surface firmly anchored to underlying basement membrane by many hemidesmosomes with abundant collagen fibers in the anterior stromal layer. Low-energy 1.5 mJ sample showed minor thermal damage at ablated regions with mild intra corneal cracks and separation of interdigitating corneal keratinocytes. Moreover, most of keratinocytes showed normal structures without abnormal morphological alterations with intact basement membrane. High-energy 2.0 mJ sample showed a significant loss, thermal damage and undulation of corneal keratinocytes with detachment from the underlying basement membrane.

### Evaluating the DNA damage

**Fig. 4:**
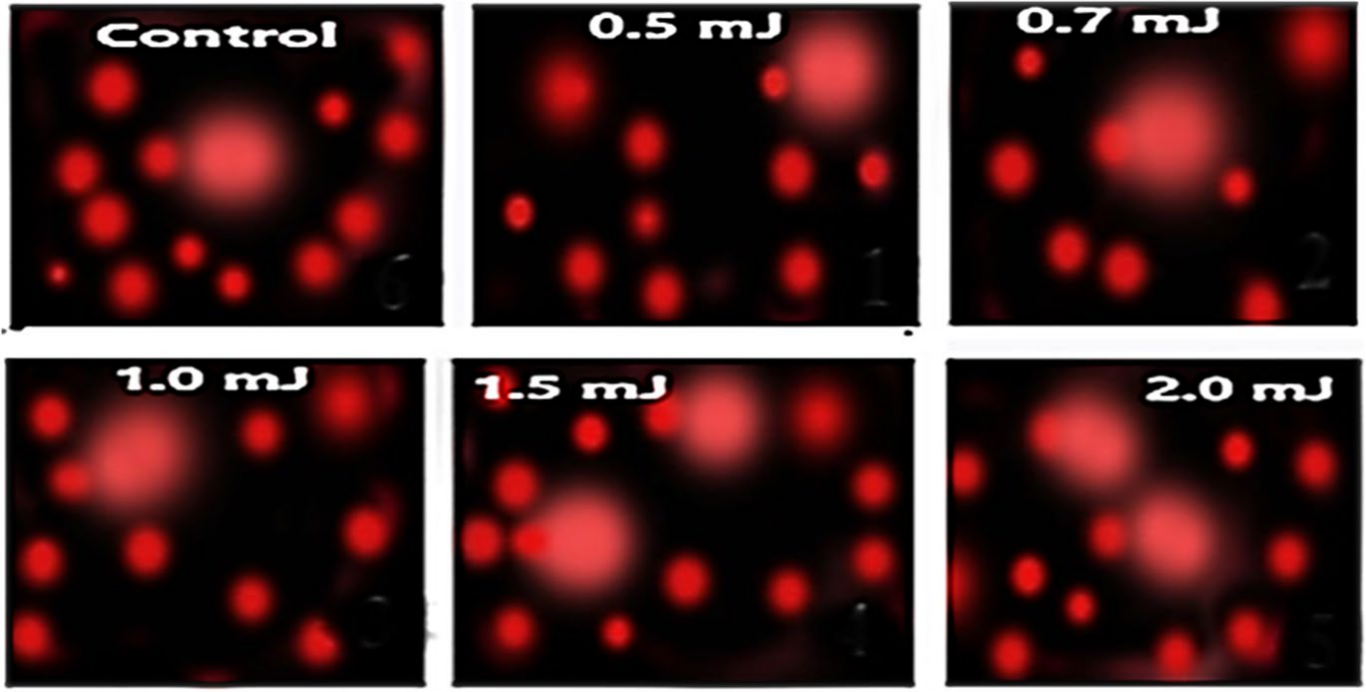
Effect of 266-nm solid-state laser with five different energies compared with the control on the corneal DNA. Scale bars: 100 µm

The comet images for the corneas DNA (Fig. 4) show the comet head contains DNA with the high molecular weight while the comet tail contains the principal ends of migrating fragments. Table 2 illustrates the percentage of tailed and untailed cells, the tail length, the percent of DNA in the tail and the tail moment. The result revealed that, for the 1.5 mJ, the percentage change in tail length was 50.5%, the percentage change in the percentage of DNA in the tail was 42.15%, and in the tail moments, the percentag changes was 114.2% with respect to the control sample, whereas, for the 2.0 mJ, the percentage change in tail length was 84.7%, the percentage change in the percentage of DNA in the tail was 58.7% and the percentage change in the tail moments was 194% with respect to the control. Moreover, the result indicated that the DNA change of the cornea exposed to 2.0 mJ is more significant than the 1.5 mJ group.

**Table 2:** Percentage of tailed and untailed cells, the tail length, the percent of DNA in tail and the tail moment after exposure to different energies of 266-nm laser

Energy (mJ)	Tailed (%)	Untailed (%)	Tail length (µm)	Tail DNA (%)	Tail moment
Control	3	97	1.11	1.21	1.34
0.5	4	96	1.27	1.31	1.66
0.7	4	96	1.33	1.41	1.88
1.0	6	94	1.47	1.33	1.96
1.5	7	93	1.67	1.72	2.87
2.0	9	91	2.05	1.92	3.94

**Fig. 5:**
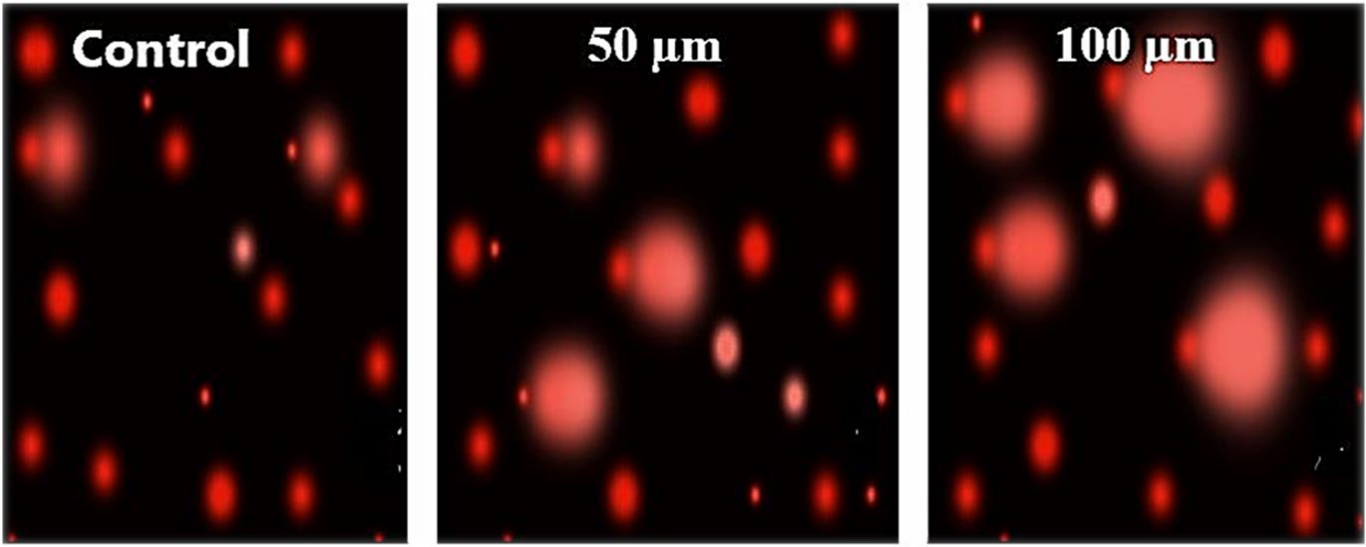
Effect of 193 nm on the corneal DNA using two different depths 50 µm, 100 µm, compared with the control. Scale bars: 100 µm

Tail moment, a measure of both amount of DNA in the tail and the distribution of DNA in the tail, became a common descriptor along with the tail length and percentage of DNA in the tail. Moreover, the moment, and the percent of DNA in the tail multiplied by the distance between the means of the head and tail distributions are all valuable measures. The comet assay results after using 193-nm laser (Fig. 5) showed a noticeable elevation in the percentage change of the DNA parameters as summarized in Table 3. The percentage change for the 50 µm depth was 124% in the tail length, 112% in the DNA% and 375% for the tail moment with respect to the control. On the other hand, the 100 µm depth sample showed a 141% change in the tail length, 169% change in DNA%.and 549% change in the tail moment. These depths were specifically studied as it is produced using the actual treatment parameters of the commercial device (MEL 80™ Excimer Laser, ZEISS, Germany). Greater depths are avoided to preserve the corneal tissue’s normal mechanics. Different parameters were used for the 193-nm laser (fluence ~ 2.6 mJ /mm^2^) since a commercial device was empolyed where the parameters could not be adjusted like they could for our produced solid-state laser (at 266 nm). The variations in the obtained results between 266 and 193 nm indicated the ability to use a 266-nm laser in the photoablation procedures.

**Table 3:** Percentage of tailed and untailed cells, the tail length, the percent of DNA in the tail and the tail moment after exposure to 193 nm

Ablation depth	Tailed (%)	Untailed (%)	Tail length (µm)	Tail DNA (%)	Tail moment
Control	4	96	1.46	1.42	2.07
50 µm	8	92	3.27	3.01	9.84
100 µm	13	87	3.52	3.82	13.44

## Conclusion

An optical setup is designed to generate 50 pulses from 266-nm wavelength laser with variable pulse energies (0.5 mJ, 0.7 mJ, 1.0 mJ, 1.5 mJ and 2.0 mJ) applied on the Ex vivo rabbit cornea with different fluences. The histological examination of the five samples revealed that the pulse energy with 1.5 mJ and 2.0 mJ showed the perfect photoablation effect with a lowest thermal effect in the 1.5 mJ than in 2.0 mJ. Moreover, the 1.5 mJ sample under the electron microscope examination showed minor thermal damage at the ablated regions with mild intra corneal cracks, while the 2.0 mJ showed significant loss and apparent thermal damage. The produced Nd:YAG (266-nm) laser beam was subjected to an extensive analysis (histological, TEM and DNA damage analysis) which was implemented and reported in detail in the current research. The ArF laser (193 nm) was a commercial device which has been extensively explored in the literature using TEM and histological analysis. Accordingly, DNA damage analysis was carried out to compare the two wavelengths since it was the primary investigation criterion in our work.

The comparison between the effect of 266 nm and 193 nm wavelengths on the DNA damage of the corneal protein through comet assay showed significant elevation in the damage parameters by using ArF excimer at 193 nm. Our finding confirms that the solid-state laser (266 nm) is an alternative to the standard gas laser (ArF excimer at 193 nm) in corneal ablation treatment. This can be achieved by selection of the best fluence that produces optimum photoablation effect with minimum thermal effect with the aid of the investigation under the light microscope, electron microscope and comet assay for the investigation of the DNA damage.

## Supplementary Information

Below is the link to the electronic supplementary material.

Supplementary file1 (PDF 207 kb)

## Data Availability

Data are available from the corresponding author upon request.
