# Supplementary material for: Evaluating the efficacy of Nd:YAG fourth harmonic (266 nm) in comparison with ArF excimer (193 nm) in laser corneal reshaping: ex vivo pilot study
Source: Int Ophthalmol. 2023 Apr 21;43(9):3087–96. doi: 10.1007/s10792-023-02708-z (PMC10400703; doi:10.1007/s10792-023-02708-z)
Supplement: Supplementary file 1 — Supplementary file1 (PDF 207 kb) [file 10792_2023_2708_MOESM1_ESM.pdf]

```
void setup() {
  pinMode(9, OUTPUT);//Shutter PIN
  pinMode(13, OUTPUT);//Arduino led pin
}
void loop() {
  delay (5000);// Delay 5 seconds before start
  digitalWrite(9,HIGH); //Open the shutter
  delay (10000);// Open shutter for 10 seconds of a laser with freqancy 10 to get 100 pulses
  digitalWrite(9,LOW); //Close the shutter
  digitalWrite(13,HIGH); //Indicator led for end of laser pulses
  delay (100000);// Wait for 100 seconds before use laser again
  digitalWrite(13,LOW); //turn off the Indicator led
}
```
